# CG7379 and ING1 suppress cancer cell invasion by maintaining cell–cell junction integrity

**DOI:** 10.1098/rsob.210077

**Published:** 2021-09-08

**Authors:** Alexandra D. Rusu, Zoe E. Cornhill, Brenda Canales Coutiño, Marcos Castellanos Uribe, Anbarasu Lourdusamy, Zsuzsa Markus, Sean T. May, Ruman Rahman, Marios Georgiou

**Affiliations:** ^1^ School of Life Sciences, University of Nottingham, Nottingham NG7 2UH, UK; ^2^ Leicester Institute for Structural and Chemical Biology, Department of Molecular and Cell Biology, University of Leicester, Leicester LE1 9HN, UK; ^3^ Department of Cell and Developmental Biology, University College London, Gower Street, London WC1E 6BT, UK; ^4^ School of Biosciences, University of Nottingham, Sutton Bonington, Leicestershire LE12 5RD, UK; ^5^ School of Medicine, Biodiscovery Institute, University of Nottingham, Nottingham NG7 2RD, UK

**Keywords:** cancer, tumour suppressor, invasion, *Drosophila melanogaster*, cell–cell junctions, ING1

## Abstract

Approximately 90% of cancer-related deaths can be attributed to a tumour's ability to spread. We have identified CG7379, the fly orthologue of human ING1, as a potent invasion suppressor. ING1 is a type II tumour suppressor with well-established roles in the transcriptional regulation of genes that control cell proliferation, response to DNA damage, oncogene-induced senescence and apoptosis. Recent work suggests a possible role for ING1 in cancer cell invasion and metastasis, but the molecular mechanism underlying this observation is lacking. Our results show that reduced expression of CG7379 promotes invasion *in vivo* in *Drosophila*, reduces the junctional localization of several adherens and septate junction components, and severely disrupts cell–cell junction architecture. Similarly, ING1 knockdown significantly enhances invasion *in vitro* and disrupts E-cadherin distribution at cell–cell junctions. A transcriptome analysis reveals that loss of ING1 affects the expression of several junctional and cytoskeletal modulators, confirming ING1 as an invasion suppressor and a key regulator of cell–cell junction integrity.

## Graphical abstract

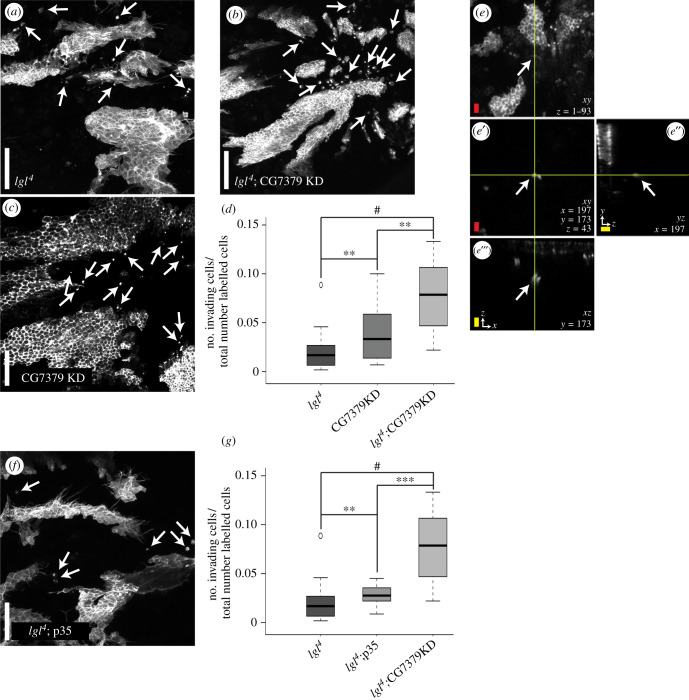

## 1. Introduction

Metastasis is the major cause of mortality in human cancers. Underlying this phenomenon are a number of highly complex cellular behaviours, whereby cancer cells must acquire an ability to invade out of the primary tumour mass, avoid anoikis, migrate directionally and disseminate to form secondary tumours at distant secondary sites. Unfortunately, the molecular mechanisms underlying these processes are poorly understood.

We have developed an *in vivo* system in *Drosophila* that allows the study of epithelial cell and tissue morphogenesis in real time [1–4]. We recently used this system to generate tumours with specific genotypes on the dorsal thorax epithelium of the fly and to screen for conserved modulators of tumour behaviour in the living animal [5]. This screen allowed the identification of numerous invasion suppressors, one of which was the *Drosophila* inhibitor of growth (ING) orthologue CG7379.

CG7379 remains largely uncharacterized in *Drosophila*. Due to the presence of conserved zinc finger and plant homeodomain (PHD)-type domains [6] CG7379 has a putative role in chromatin remodelling and the transcriptional regulation of target genes. The human orthologue of CG7379 is ING1 and other ING family members [7]. In mammals, there are five members of the ING family of proteins (ING1-5, including several splice variants), and virtually all members of this family have been shown to possess tumour suppressive functions [8]. All ING family members contain the signature C-terminal PHD finger domain. This highly conserved domain has the highest degree of homology among the ING proteins [6] and also shares a very strong identity of 78% with the PHD domain of CG7379 [7].

Loss of chromosomal locus, translocation to the mitochondria, mutation (rarely), but mainly decreased expression of ING1 have been documented in various mammalian cancers [9,10]. Several studies investigating the effect of ING1 on cell proliferation, reported cells accumulating in G0/G1 upon ING1b overexpression in a range of cell lines from normal fibroblasts to cancer cells derived from metastatic sites [11–14]. ING1 is a key player in multiple DNA repair pathways [15–17], with evidence suggesting that ING1 can induce apoptosis in response to DNA damage [18,19]. The antiproliferative and proapoptotic effects of ING1 were linked to its ability to modulate transcription. ING1 physically interacts with protein complexes with histone acetyltransferase (HAT) and histone deacetylase (HDAC) activity [19]. ING1b is a stable component of the Sin3A/HDAC 1 and 2 protein complexes and can additionally interact with subunits of the Brg1-based Swi/Snf chromatin remodelling complex [20,21], with proliferating cell nuclear antigen and p300 [19]. Additionally, ING1 has been shown to modulate the expression of several micro RNAs, which in turn further regulate gene expression [12,22].

Several studies have also associated ING family proteins with invasion and metastasis. For example, the reduced expression of ING1b and ING4 has been observed in metastatic melanoma [23,24] and reduced ING4 expression could be correlated with the human gastric adenocarcinoma stage [25]. Low levels of ING1 are associated with increased motility, migration or invasion in colorectal, gastric and breast cancers [26–28]. It was further shown that ING4 overexpression inhibited melanoma cell migration and invasion *in vitro* [24]. ING1 overexpression was also shown to inhibit cell migration, invasion and metastasis both *in vitro* and *in vivo* [14,28], and ING1 knockdown in MDA-MB-231 cells increased migration and invasion [28]. However, despite ING family proteins being repeatedly implicated in tumour progression and invasion, little is known of the molecular mechanisms that underlie these tumour suppressive effects. Here, using two independent RNAi lines against the *Drosophila* ING orthologue CG7379, we demonstrate that loss of this protein results in a severe disruption to the adherens (AJ) and septate junctions (SJ), resulting in a loss of epithelial integrity and increased invasion. We further show that the loss of ING1 in human cancer cells also promotes invasion and disrupts cell–cell adhesion, through altered gene expression.

## 2. Results

### 2.1. CG7379 acts as an invasion suppressor

We recently carried out an *in vivo* large-scale screen for genes that affect tumour behaviour by (i) generating positively marked clones on the dorsal thorax of the fly that are homozygous mutant for the neoplastic tumour suppressor gene *lethal (2) giant larvae (lgl^4^)*; (ii) specifically labelling the mutant tissue with GFP:Moe (the actin-binding domain of moesin fused to GFP), thereby labelling the actin cytoskeleton of these cells; (iii) overexpressing an RNAi transgene to deplete expression of a gene of interest specifically within the mutant, labelled tissue. In this way, we could identify genes that when knocked down (KD) work cooperatively with *lgl^4^* to promote tumour progression [5].

**Figure 1.**
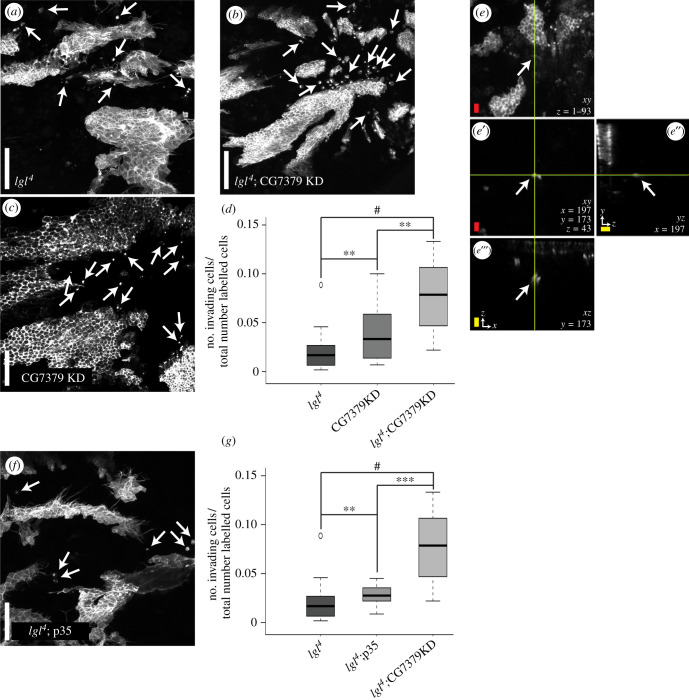
CG7379 KD promotes invasion. (*a–d*) GFP:Moe labelled clones mutant for (*a*) *lgl^4^*, (*b*) *lgl^4^*;CG7379KD and (*c*) CG7379KD. CG7379KD results in a highly invasive phenotype, which is enhanced when accompanied by a *lgl^4^* mutation. (*d*) Invading cells were identified based on their characteristic round shape and based on their ability to migrate away from the clone within the tissue plane and/or extrude basally from the dorsal epithelium. The number of invading cells were counted and divided by the number of GFP-labelled cells within the field of view. (*e*) *z* max intensity projection of the back of a 28 h APF (after puparium formation) old *Drosophila* pupa carrying GFP:Moe labelled CG7379KD clones. (*e*′) Individual confocal *z* slice highlighting a GFP:Moe labelled cell located 25 µm beneath the epithelial sheet. *z* = confocal slice; *x* and *y* = coordinates and position of the invasive cell. (*e*″) *yz* orthogonal view projection of the invasive cell in (*e*′). (*e*″′) *xz* orthogonal view projection of the invasive cell in (*e*′). (*f–g*) *lgl^4^*; p35 (*f*) does not phenocopy *lgl^4^*; CG7379KD (*b*). Although a significant increase in invading cells is observed when overexpressing p35 within *lgl^4^* clones, this increase is significantly lower than that observed in *lgl^4^*; CG7379KD clones (quantified in g). *lgl^4^ *n** = 42, CG7379KD *n* = 21, *lgl*;CG7379KD *n* = 21, *lgl*;p35 *n* = 21). Kruskal–Wallis test was performed to determine statistical significance. White scale bar: 50 µm, red scale bar: 10 µm, yellow scale bar: 10 µm in the *z* plane. ^#^*p* < 0.0001; ***p < 0.001; **p < 0.01; *p < 0.05.

Our screen identified CG7379 as a strong hit for invasion, with *lgl^4^*; CG7379KD mutant clones showing a significant increase in the number of polarized epithelial cells seen beneath the epithelial sheet [5] (figure 1*a–e*). As well as promoting frequent cell delamination, we found *lgl^4^*; CG7379KD clones to be multilayered and to promote abnormal protrusion morphology (electronic supplementary material, figure S1).

GFP:Moe labelled CG7379KD clones, without the *lgl^4^* mutation, formed an organized monolayered epithelium with significantly fewer invading cells found beneath the epithelium when compared to *lgl^4^*; CG7379KD mutant clones (figure 1*b–d*). However, there was also a significant increase in invasion when compared to *lgl^4^* mutant clones (figure 1*d*). It therefore appears that although CG7379KD promotes epithelial cell delamination, there is a strong cooperative effect when this KD is accompanied by a loss of lgl function, leading to increased multilayering and invasion.

### 2.2. Increased invasion following CG7379KD cannot be solely explained by cell death evasion

It is known that ING1 can act as a tumour suppressor by inducing apoptosis in damaged cells [19,29]. It is therefore possible that the invasive phenotype that we observe when knocking down CG7379 in the *Drosophila* notum could be due to the increased survival of invading cells, whereby invading cells are able to evade elimination via apoptosis. To test this, we overexpressed P35 specifically within GFP:Moe labelled *lgl^4^* mutant clones. The overexpression of P35 is known to prevent apoptotic death in a wide variety of tissues and is widely used in *Drosophila* cell death studies [30]. If the increase in invasion observed in *lgl^4^*; CG7379KD mutant clones was largely due to an inhibition of apoptosis; we would expect to see a similar increase in GFP:Moe positive cells beneath the epithelial sheet when expressing P35 in *lgl^4^* mutant clones. This however was not observed. Although a significant increase in GFP:Moe positive cells was observed when comparing *lgl^4^* mutant clones with *lgl^4^*; P35 clones (*p* = 0.009), we did not see the dramatic increase in the number of invasive cells that we observe in *lgl^4^*; CG7379KD clones (figure 1*f,g*).

**Figure 2.**
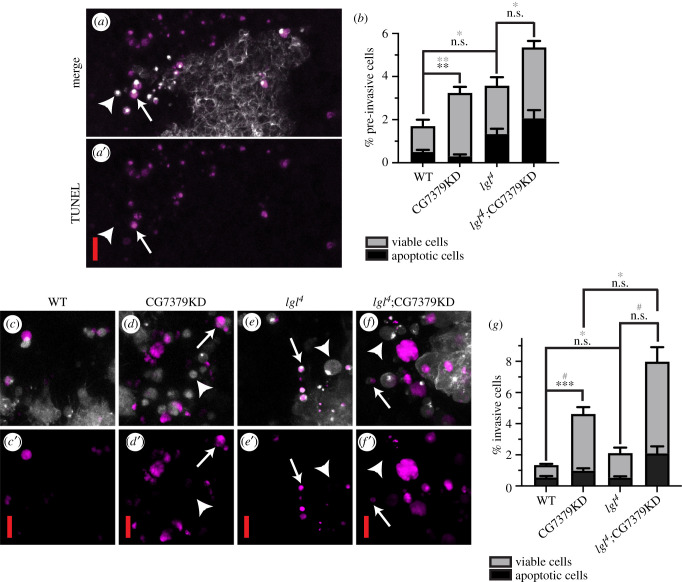
CG7379KD promotes evasion of apoptosis. (*a*) A TUNEL (magenta) and GFP:Moe (white) labelled *lgl^4^* mutant clone, highlighting both apoptotic (arrow) and non-apoptotic (arrowhead) pre-invasive cells. (*b*) Quantification of the proportion of apoptotic pre-invasive cells (black), as determined by TUNEL and c-Dcp-1 staining. Grey, % pre-invading cells/total number of labelled cells; black, % apoptotic pre-invading cells/total number of labelled cells. When comparing CG7379KD with WT clones, significantly more pre-invasive cells (grey asterisks) were observed, out of which significantly fewer were apoptotic (black asterisks) (*n* ≥ 138 cells across 10 animals/genotype). (*c–f*) Example images of apoptotic (arrow) and non-apoptotic (arrowhead) invasive cells. iCasper (magenta) and Moe:GFP (white) labelled mutant clones (genotypes specified above panels). Images represent *z* stack projections. (*g*) Quantification of (*c–f*). Grey, % invading cells/total number of labelled cells; black, % apoptotic invading cells/total number of labelled cells. *n* ≥ 346 cells across 20 animals/genotype. Kruskall–Wallis test was performed to determine statistical significance. Error bars represent ± s.e.m. Scale bars: 10 µm. ^#^*p* < 0.0001; ***p < 0.001; **p < 0.01; *p < 0.05.

We additionally looked for apoptosis within pre-invasive cells, using both a TUNEL assay and an anti-cleaved *Drosophila* Dcp-1 antibody (figure 2*a,b*). Pre-invasive cells are cells that are about to delaminate from the epithelial sheet and can be identified by their rounded morphology and the presence of a characteristic actin-rich spot at one side of the cell prior to invasion [5]. CG7379KD simultaneously significantly increased the number of pre-invasive cells present within a clone and also reduced the amount of apoptosis occurring within these pre-invasive cells, when compared to WT clones. However, when comparing *lgl^4^*; CG7379KD clones with *lgl^4^* clones, no effect on the proportion of apoptotic pre-invasive cells was observed (figure 2*b*). This suggests that although CG7379KD probably plays a role in preventing apoptosis, the significant increase in the number of invading cells following CG7379KD is unlikely to be only due to cell death evasion.

The viability of cells once they had delaminated from the epithelial sheet was next assessed using the *in vivo* caspase sensor, iCasper (figure 2*c–g*). Activation of this construct was previously reported to reflect induction of apoptosis. Moreover, the fluorescent signal has been shown to label all stages of apoptosis starting from cell rounding and persisting even after phagocytosis of the apoptotic cell [31]. We found the proportion of apoptotic invasive cells was significantly reduced by CG7379KD when compared to WT clones. However, although *lgl^4^*; CG7379KD dramatically increases the number of viable invading cells when compared to *lgl^4^* clones, no significant reduction in the proportion of apoptotic invasive cells was observed (figure 2*g*). These results provide further evidence to suggest that the cooperative effect observed between the *lgl^4^* and CG7379KD mutations is not simply due to an increase in cell viability.

### 2.3. CG7379 is required to maintain epithelial cell–cell junction integrity

Reduced ING expression has been previously observed in high-grade metastatic cancers [23–25]. However, despite ING family proteins being implicated in invasion and metastasis, little is known of the molecular mechanisms that underlie these effects.

**Figure 3.**
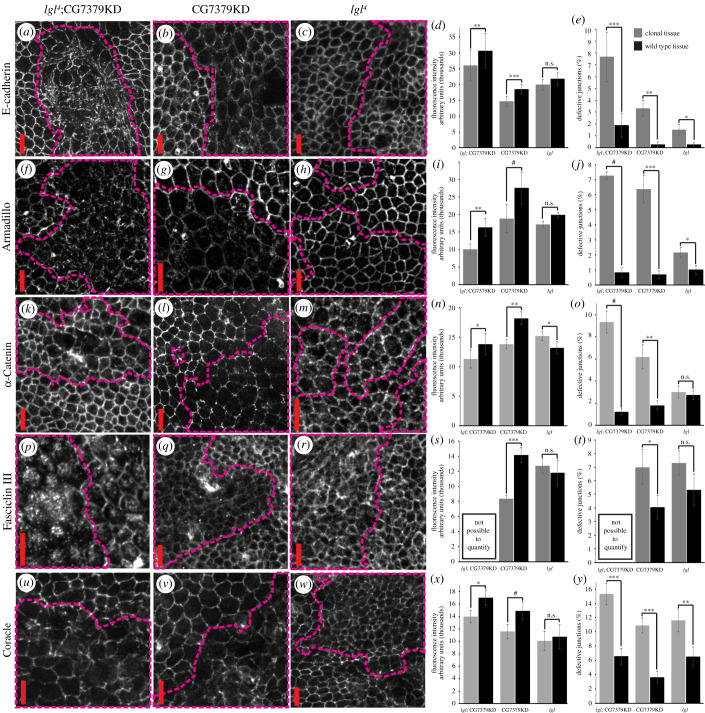
CG7379KD disrupts cell–cell junction integrity. *Drosophila* pupal nota containing positively marked mutant clones for *lgl^4^*;CG7379KD; CG7379KD alone; or *lgl^4^* alone (highlighted by dashed lines). Nota were stained for the following junctional proteins: (*a–c*) E-cadherin, (*f–h*) Armadillo (β-catenin), (*k–m*) α-Catenin, (*p–r*) Fasciclin III, (*u–w*) coracle. A severe disruption in junction protein localization and junction integrity was observed in *lgl*; CG7379KD and CG7379KD clones, as quantified in panels (*d,e*; *i,j*; *n–o*; *s,t*; *x,y*) (*n* ≥ 65 junctions from a minimum of nine animals for each genotype). Fasciclin III staining could not be quantified in *lgl*; CG7379KD clones due to an almost complete lack of protein localization at the cell cortex. Scale bars = 10 µm. Error bars represent ± s.e.m. Student's *t*-test was performed to determine statistical significance. ^#^*p* < 0.0001; ***p < 0.001; **p < 0.01; *p < 0.05.

Our screen identified *lgl^4^*;CG7379KD as affecting epithelial architecture, protrusion morphology and promoting frequent cell delamination [5] (figure 1; electronic supplementary material, figure S1). These phenotypes therefore implicate effects on adhesion, polarity and actin regulation as possible underlying influences on the observed cell behaviour. To investigate the molecular mechanisms by which loss of CG7379 expression could affect epithelial cell organization, we first investigated cell–cell adhesion using antibodies to proteins that localize to either the AJ or SJ (the functional equivalent of tight junctions in insects). We stained for E-cadherin, Armadillo (β-catenin), and α-catenin (AJ proteins), and Fasciclin III, Coracle and Discs Large (SJ proteins) (figure 3; electronic supplementary material, figure S2). We generated positively marked mutant clones, surrounded by wild-type tissue, thereby allowing us to directly compare junction composition inside and outside mutant clones in the same tissue. We looked in mutant clones for *lgl^4^*;CG7379KD, CG7379KD alone and *lgl^4^* alone. The protein level was estimated based on the level of fluorescence intensity at the cell–cell interface. Intact cell junctions were randomly chosen for analysis within the clonal or WT tissue. Cells at the boundary between the clonal and WT tissue were excluded from the analysis since it has previously been shown that their morphology can be influenced by the neighbouring WT cells [32]. The percentage of defective junctions was calculated as the ratio of missing and/or fragmented cell junctions identified per total number of junctions analysed. In most cases, AJ and SJ protein localization was significantly reduced and appeared fragmented and poorly distributed along cell junctions in both *lgl^4^*;CG7379KD and CG7379KD clones, with minor effects observed in *lgl^4^* clones (figure 3). The only exception was when staining for Dlg, where the most severe effects were observed in *lgl^4^* mutant clones (electronic supplementary material, figure S2a–d). It has been previously shown that such a fragmented distribution of E-cadherin, Armadillo and α-catenin correlates with junctional discontinuities [1]. Our data therefore suggest that AJ and SJ integrity was significantly and severely disrupted by CG7379KD.

We next looked at whether the apicobasal polarity determinants aPKC, bazooka (Par3) or Crumbs were affected in these mutant clones, as a disruption of apicobasal polarity frequently promotes invasion. We only found a significant effect on aPKC localization, which is probably due to a loss of lgl function, as no significant effect was found in CG7379KD clones (electronic supplementary material, figure S2). The additional effects on cell polarity in *lgl^4^* mutant clones (i.e. the mislocalization of both Dlg and aPKC) probably explain the increased multilayering and invasion observed in *lgl^4^*;CG7379KD clones, when compared with CG7379KD alone.

Since E-cadherin is at the core of cell–cell junction adhesion [33] and since E-cadherin localization at the AJ was severely disrupted in both *lgl^4^*;CG7379KD and CG7379KD clones, we hypothesized that if E-cadherin levels could be restored, this may partially rescue cell–cell junction defects and consequently cell delamination. Therefore, we attempted to rescue the CG7379KD invasive phenotype by overexpressing E-cadherin under the Ubi-p63E promoter in CG7379KD mosaic animals (electronic supplementary material, figure S3). The construct we used has been shown to substitute for a null *shotgun* allele (the gene encoding E-cadherin in flies) and to function and behave normally in the absence of intact E-cadherin [34]. We however found no significant effect on invasion, suggesting that either (i) E-cadherin overexpression is not sufficient to compensate for the CG7379KD-mediated disruption to multiple AJ and SJ junction components or (ii) E-cadherin mis-localization to the AJ, rather than simple expression levels, may be responsible for the observed phenotype.

### 2.4. ING1 regulates cell adhesion through transcriptional regulation of cell adhesion modulators

**Figure 4.**
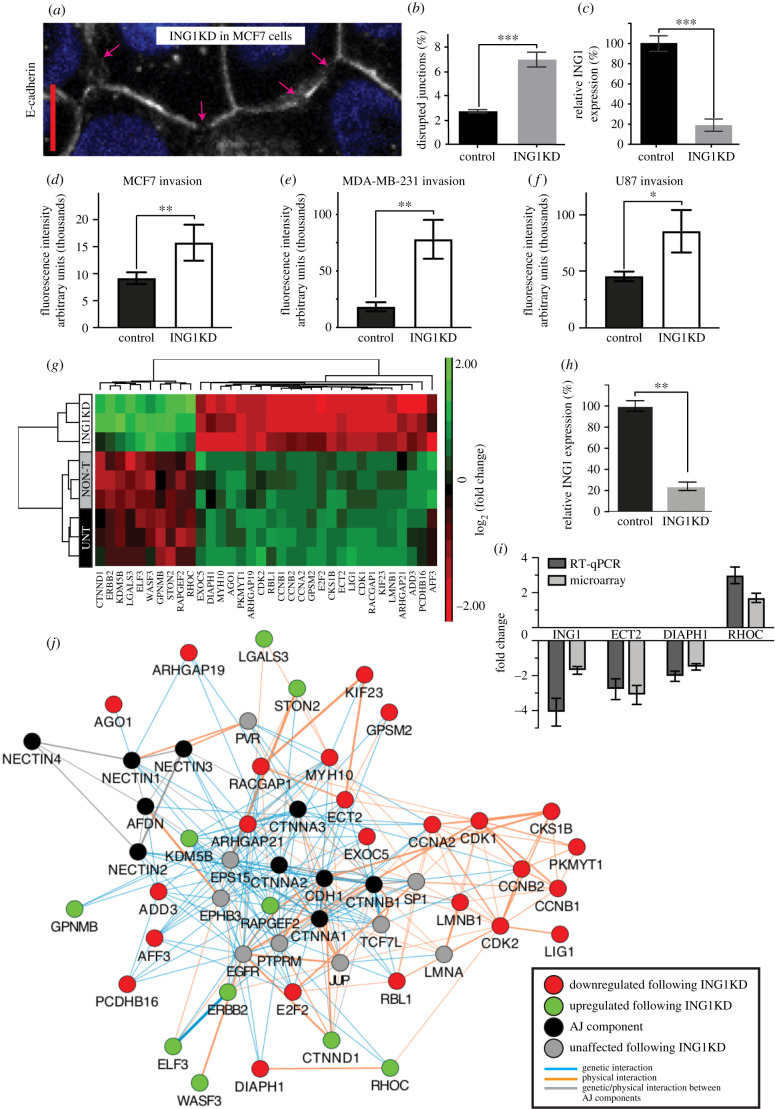
ING1KD increases the invasive potential of several cancer cell lines and disrupts cell–cell junction integrity in MCF7 cells. (*a–c*) MCF7 cells transfected with siRNA for ING1 and stained for E-cadherin. (*a*) Disruption to E-cadherin localization at the junction was observed (arrows highlight junctional breaks). Quantification shows a significant increase in disrupted junctions upon ING1KD. (*b*) NON-T MCF7 cells were used as a control. *n* = 606 junctions for ING1KD and 823 junctions for the control. Relative ING1 mRNA levels, determined by qRT-PCR (*c*). (*d–f*) Transwell invasion assays for MCF7 (*d*), MDA-MB-231 (*e*) and U87 cells (*f*). In order to invade, cells had to pass through an 8 µm pore membrane coated with Matrigel. A significant increase in invasion for all three cell lines was observed following ING1KD in comparison to NON-T cells. (*g*) Heat map representation of unsupervised clustering of 34 selected DEG following ING1KD in MCF7 cells. Columns represent genes, each row represents a sample. Treatment groups are specified on the left-hand side. Colour code represents log2 of the fold change of expression: red, downregulated; green, upregulated. Horizontal and vertical clusters were created based on Euclidean distance. (*h*) Relative ING1 mRNA levels, determined by qRT-PCR, for transcriptomics experiment. (*i*) Bar chart comparing microarray and RT-qPCR for four genes with differential expression between ING1KD cells and NON-T cells. (*j*) Interaction network showing physical and genetic interactions between selected DEG by ING1KD and proteins associated with the AJ. The GeneMANIA plug-in for Cytoscape was used to generate an interaction network based on previously documented interactions. Black nodes mark AJ components, red nodes: genes that were downregulated by ING1KD; green nodes: genes that were upregulated by ING1KD; grey nodes: genes through which they interact (standard settings: max resultant attributes = 10; max resultant genes = 20); orange lines: physical interactions; blue lines: genetic interactions; grey lines genetic or physical interactions between AJ components. Line thickness is indicative of the score (weighting: GO molecular function). Scale bar = 10 µm. Error bars represent ± s.e.m. Student's *t*-test was performed to determine statistical significance. ^#^*p* < 0.0001; ***p < 0.001; **p < 0.01; *p < 0.05.

Having identified CG7379 as an important invasion suppressor, we wanted to test whether its closest human orthologue, ING1, would also act in a similar way. Using an antibody against human E-cadherin on the breast cancer cell line MCF7, we found a significant disruption to both E-cadherin localization and to AJ integrity following ING1KD, with frequent junctional breaks observed (figure 4*a–c*), suggesting that human ING1 also plays an important role in maintaining AJ integrity. We recently demonstrated that KD of ING1 led to an increase in migration and invasion in MCF7 cells, using an *in vitro* invasion assay [5]. Here, we additionally show that this effect on invasion is not restricted to MCF7 cells, as ING1KD also increases invasion in U87 (derived from malignant glioma) and MDA-MB-231 cells (derived from malignant breast adenocarcinoma) (figure 4*d–f*). Relative ING1 mRNA levels following KD, as determined by qRT-PCR were 28% (MCF7), 4% (MDA-MB-231) and 9% (U87) (data not shown). In line with these results, ING1KD has previously been shown to increase migration and invasion in MDA-MB-231 cells [28].

ING1 is known to act as both a transcriptional activator and repressor through interactions with DNA and a variety of epigenetic regulatory proteins [35]. In an effort to understand how the loss of ING1 function might lead to a disruption to cell–cell junctions we performed a microarray gene expression analysis. We analysed and compared gene expression in MCF7 cells post ING1KD with untreated cells and cells treated with non-targeting siRNA (NON-T). Out of 21448 genes analysed, the expression of 919 genes was significantly altered as a result of ING1KD (FDR < 0.05, *p* < 0.01, FC ≥ 1.5 or FC ≤ −1.5; electronic supplementary material, table S1 and figure S4). Gene ontology (GO)-enrichment analysis and pathway analysis showed that most of the significantly altered genes are involved in cell cycle regulation and DNA replication (electronic supplementary material, table S2) which corroborates previous work on ING1 function [9]. However, we additionally found 34 differentially expressed genes (DEGs) that are likely to affect both cell–cell junction integrity and promote invasive phenotypes, including DIAPH1, ECT2, EXOC5, WASF3, RAPGEF2, ADD3, KDM5B, CTNND1 and RHOC (figure 4*g*). There is extensive evidence linking the misregulation of these genes with a disruption to cell–cell junction integrity, and to the promotion of invasion (see Discussion). We additionally used RT-qPCR on a selection of genes (RHOC, DIAPH1, ECT2 and ING1) to verify the microarray results, with qPCR showing a similar or greater expression fold change in all cases (figure 4*i*); results from the two analyses show a high correlation (Pearson test: *r* = 0.89, *p* = 0.05).

The Cytoscape plug-in GeneMANIA [36] was used to assess interactions between the selected subset of 34 DEGs and AJ components. For each selected DEG, we searched for physical or genetic interactions, validated by experimental data, including yeast two-hybrid, co-immunoprecipitation and other interaction data from various databases (see Methods). The resultant network (figure 4*j*; electronic supplementary material, data S1) reveals a total of 141 genetic interactions and 116 physical interactions between 34 DEGs and 10 AJ KEGG pathway genes (electronic supplementary material, table S3) indicating that the DEGs in ING1KD cells extensively interact with members of the AJ pathway.

## 3. Discussion

The *in vivo* model for epithelial cancer that we have developed has proven to be particularly suitable for the study of cancer cell invasion. Major advantages of our system include our ability to generate tumours surrounded by wild-type tissue and the native local microenvironment, thereby maintaining the complex tumour–stroma interactions known to influence tumour behaviour [37], and the ability to image tumour behaviour in real time in the living animal. This has led to the identification of numerous invasion suppressors [5], including CG7379, whose KD very strongly promotes invasion from the dorsal thorax epithelium. Although CG7379 is largely uncharacterized in *Drosophila*, it has been picked up in numerous screens, focusing on diverse biological functions, including Notch pathway regulation, muscle morphogenesis, adiposity regulation and airway morphogenesis [38–42]. The latter screen is important as it implicates CG7379 in the maintenance of epithelial integrity in the tubes that make up the fly tracheal system.

The human orthologues of CG7379, the ING family proteins, have been extensively implicated in invasion and metastasis but little was known of the molecular mechanisms that underlie these effects. Here, using the *Drosophila* ING orthologue CG7379, we demonstrate that the loss of this protein results in a severe disruption to both the AJ and SJ, resulting in a loss of epithelial integrity and increased invasion. For instance, E-cadherin and Armadillo, whose expression levels were severely reduced by CG7379KD, are often differentially expressed in human cancers, which often correlate with increases in invasion [43]. Fasciclin III, whose distribution at the SJ was most affected in *lgl^4^*;CG7379KD clones, promotes homophilic interactions between cells, enhancing cell–cell adhesion. Changes in fasciclin III expression alone can be sufficient to alter intercellular adhesion [44]. Moreover, fasciclin-like proteins are known to be associated with metastasis in human cancers [45,46]. Coracle expression and distribution at the SJ was also affected by CG7379KD. As a member of the Yrt/Cora-group of proteins, coracle may act towards stabilizing the lateral actin cytoskeleton [47,48], which in turn acts on cell–cell junction stability [33].

Despite the fact that E-cadherin plays a central role in cell–cell junction formation and maintenance [33], ubiquitous overexpression of E-cadherin in the fly did not significantly affect the level of delamination caused by CG7379 KD. Junctional discontinuities were previously observed in the *Drosophila* pupal notum defective for E-cadherin turnover [1], which suggests a possible role for CG7379 in the regulation of E-cadherin localization and/or turnover. Our inability to rescue the increase in invasion by reintroducing E-cadherin into the system is consistent with other *in vitro* studies. These studies found that the localization of E-cadherin to cell junctions is under the control of Ras, and artificial increase of E-cadherin levels without manipulation of Ras expression leads to cytoplasmic accumulation of the overexpressed protein [49,50].

Our data indicate that CG7379KD may not have a drastic effect on apicobasal polarity as in our experiments, aPKC, bazooka and crumbs localize correctly to the cortex following CG7379KD. However, the cooperative effect between CG7379KD and *lgl^4^* does lead to more accentuated defects at cell–cell junctions, including more profound defects in dlg localization, as well as the additional mis-localization of aPKC. This disruption to polarity could explain the significant increase in invasion that is observed in *lgl^4^*;CG7379KD clones when compared to CG7379KD clones. We do think it likely, however, that the cooperation between CG7379 and *lgl^4^* extends beyond the role that lgl plays in cell polarity. It is known that *lgl^4^* promotes cell transformation in the epithelium [51,52]. In addition to its role as a key polarity protein, lgl influences the actin cytoskeleton, regulates exocytosis and can also interact with other non-polarity proteins and subsequently act in Hippo, Wingless-related integration site (Wnt) and Notch signalling, being therefore involved in the regulation of several biological processes, such as cell cycle progression and apoptosis [53].

Our results further suggest that the loss of CG7379 leads to an avoidance of anoikis following cell detachment from the epithelial sheet, demonstrating the multiple tumour and invasion suppressive roles of this gene. It has been previously shown that cells from the notum of the fly can delaminate basally and then undergo a process similar to anoikis within 10 min of delamination [54]. We have imaged invading cells in our system for up to 2 h post-delamination without observing any obvious signs of cell death. Moreover, the invasion patterns observed in our experiments are phenotypically different to those described by Marinari *et al*. as cells delaminate irrespective of their position within the epithelium, as opposed to preferentially at the midline. Moreover, most invading cells were viable at the time of analysis, with CG7379KD significantly decreasing the number of invasive cells that activate executioner caspases. This suggests that invading cells do not undergo immediate cell death and is in line with the ability of ING1, the CG7379 human orthologue, to modulate apoptotic processes [10,19,55,56].

Our transcriptomics experiments, knocking down ING1 expression in MCF7 breast cancer cells, provide some mechanistic insight into how the loss of ING1 function might affect cell–cell junctions and promote invasion. The expressions of numerous genes that regulate cell–cell adhesion and/or the actin cytoskeleton were altered following ING1KD in MCF7 cells. This included EXOC5, which was significantly downregulated and shows high homology to *Drosophila* sec10. Sec10 has been shown to interact with Armadillo (β-catenin) and α-Catenin [57] and via sec5 and 15 can reduce E-cadherin, Armadillo and α-Catenin levels at AJs in *Drosophila* [57]. Furthermore, increased EXOC5 mRNA levels were previously correlated with E-cadherin overexpression within the lymphovascular embolus of inflammatory breast cancer [58].

RHOC and KDM5B were both significantly upregulated in our study. RHOC has been shown to be a marker of metastatic potential in some breast cancers [59]. RHOC overexpression has been associated with increased cell motility and invasion through stress fibre formation and focal adhesion, while its KD has been shown to decrease invasion and nuclear β-catenin levels [59–61]. An increase in RHOC expression was reported to induce a decrease in E-cadherin levels at the AJ in MCF7 cells (but not overall E-cadherin levels) without affecting Snail nor Twist levels nor promoting nuclear β-Catenin accumulation [62]. These results fit both our transcriptomics results and the effect we observe on junction integrity in *Drosophila* nota when knocking down CG7379 expression.

In breast cancer, KDM5B and E-cadherin present reverse patterns of expression [63]. Moreover, the ectopic expression of KDM5B has been shown to promote EMT by downregulating E-cadherin through SNAIL independent mechanisms [63]. Overexpression of KDM5B can also increase invasion *in vitro* and metastatic potential of gastric tumours *in vivo*, by activating the Akt pathway. KDM5B is therefore very likely to modulate cytoskeleton dynamics [64]. In *Drosophila* haemocytes, little imaginal discs (lid), the fly orthologue of KDM5B, modulates Rac and Ras expression and consequently actin cytoskeleton organization. Lid depletion has been shown to trigger lamellipodia formation and hyper-polymerization of F-actin [65].

Adducin 3 (ADD3) expression was twofold decreased in our study. Adducin proteins are critical for proper formation and stabilization of the membrane cytoskeleton and regulation of cell motility and cell–cell adhesion [66]. ADD3 depletion was shown to negatively affect both AJ and tight junction reassembly and to impair the assembly of actin filaments associated with newly formed junctions in colon epithelial cells [67]. Similarly, in *Drosophila* embryos, hu-li tai shao (hts), the fly orthologue of human adducins, was shown to partially colocalize with dlg and to regulate dlg targeting to the membrane. Moreover, *hts* mutants displayed phenotypes indicative of disruptions to epithelial integrity (decreased cuticle secretion in embryos) [68]. In *Drosophila* oocytes, improper localization of *hts* RNA led to the overgrowth of actin filaments [69].

DIAPH1, a downstream target of RHOA, was downregulated by ING1KD. Its loss has been associated with a decrease in the localization of E-cadherin, α- and β-catenin at AJs, and also affected tight junction composition and junctional actin levels [70]. Additionally, ING1KD also affected the expression levels of several regulators of Rho GTPase activity, including ECT2, a RhoGEF that is required to activate Rho signalling at the zonula adherens and support junctional integrity through myosin IIA [71].

In line with a potential involvement of ING1 in cytoskeletal dynamics, the genetic screen for modulators of tumour progression, previously performed in our laboratory, has indeed highlighted CG7379 as a hit for a number of cytoskeleton relevant phenotypic categories, such as protrusion length, thickness and branching. Moreover, pre-invasive cells delaminating from CG7379KD tissues display a characteristic actin-rich spot as previously described [5]. Dynamic remodelling of the actin cytoskeleton is essential for cell motility and migration and actin polarization together with actin-myosin contractions are responsible for generating the force required for migration in both normal and pathological conditions [72–74].

Our transcriptomics results therefore confirm ING1 as an invasion suppressor that plays an important role in maintaining junction stability, and as a modulator of cytoskeletal dynamics. This perfectly recapitulates results from the fly, where CG7379KD strongly promotes invasion, severely disrupts cell–cell junction integrity and affects protrusion morphology. The ING family of tumour suppressors is well known to suppress tumourigenesis through the regulation of cell cycle progression, apoptosis and DNA repair. This work, by recognizing CG7379 and ING1 as invasion suppressors, adds further insight into the multiple roles that collectively perform ING's tumour suppressive function.

## 4. Methods

### 4.1. Transgenic *Drosophila* stocks and crosses

Fly stocks were raised on standard medium at 18°C and crosses on standard medium with yeast at 25°C. The following stocks were used: yw, neoFRT19A (Chr X; #1744); UAS-p35 (Chr 2; #6298); sna[Sco]/CyO; UAS-iCasper-noGFP-T2A-HO1}VK00005/TM6B (Chr 2,3; #64186) obtained from Bloomington Drosophila Stock Center (Indiana, USA); P{GD12222}v27988 (Chr3; #27988); P{GD12222}v27989 (Chr 3; #27989) from VDRC stock centre (Vienna, Austria); Ubi-p63E-shg:GFP (Chr 2, #109007) from KYOTO Stock Center (DGRC).

Ubx-FLP; neoFRT40A/Cyo-GFP; Pnr-GAL4, UAS-GFP:Moe/TM6b;
Ubx-FLP; neoFRT40A, *lgl[4]*/Cyo-GFP; Pnr-GAL4, UAS-GFP:Moe/TM6b;
w; tub-Gal80, neoFRT40A; MKRS/TM6b;
w; IF/Cyo-GFP; Pnr-GAL4, UAS-GFP:Moe/TM6B;
Ubx-FLP, neoFRT19A, tub-GAL80; MKRS/TM6B were laboratory stocks.

We recombined UAS-iCasper-noGFP-T2A-HO1}VK00005 and P{GD12222}v27988 or P{GD12222}v27989 on the 3^rd^ chromosome to generate new stocks.

The following genotypes were imaged. For WT clones: Ubx-FLP/+; neoFRT40A/tub-GAL80, FRT40A; Pnr-GAl4, UAS-GFP:Moe/MKRS and Ubx-Flp/+; neoFRT40A/tubGAL80, neoFRT40A; PnrGal4, UAS-GFP:Moe/UAS-iCasper.

For *lgl^4^* mutants: Ubx-FLP/+; neoFRT40A, *lgl[4]*/tub-GAL80, neoFRT40A; Pnr-GAl4, UAS-GFP:Moe/MKRS and Ubx-Flp/+; neoFRT40A, *lgl[4]*/tub-GAL80, neoFRT40A; PnrGal4, UAS-GFP:Moe/UAS-iCasper.

For p35 overexpression: Ubx-FLP/+; neoFRT40A, *lgl[4]*/tub-GAL80, neoFRT40A; Pnr-GAl4, UAS-GFP:Moe/UAS-p35.

For CG7379 knockdown: Ubx-FLP/+; neoFRT40A/tub-GAL80, neoFRT40A; Pnr-GAl4, UAS-GFP:Moe/UAS-RNAi and Ubx-Flp/+; neoFRT40A/tub-GAL80, neoFRT40A; PnrGal4, UAS-GFP:Moe/UAS-RNAi, UAS-iCasper and Ubx-Flp, tub-GAL80, neoFRT19A/ neoFRT19A; PnrGal4, UAS-GFP:Moe/UAS-RNAi.

For CG7379 KD in *lgl^4^* mutant clones: Ubx-FLP/+; neoFRT40A, *lgl[4]*/tub-GAL80, neoFRT40A; Pnr-GAl4, UAS-GFP:Moe/UAS-RNAi and Ubx-Flp/+; neoFRT40A, *lgl[4]*/tub-GAL80, neoFRT40A; PnrGal4, UAS-GFP:Moe/UAS-RNAi, UAS-iCasper.

For E-cadherin overexpression: neoFRT19A/ Ubx-FLP, neoFRT19A, tub-GAL80; +/Ubi-p63E-shg:GFP; Pnr-GAL4, UAS-GFP:Moe/UAS-RNAi.

### 4.2. Cell stocks and maintenance

MDA-MB-231 cells were obtained from Dr Sally Wheatley, U87 cells from Dr Ruman Rahman and MCF7 cells from Dr Anna Grabowska (Faculty of Medicine & Health Sciences, University of Nottingham). Cells were cultured in DMEM (MDA-MB-231 and U87) or RPMI medium without phenol red (MCF7) (Invitrogen) supplemented with 2 mM L-glu (Sigma-Aldrich) and 10% fetal bovine serum (Sigma-Aldrich) and grown in T75 culture flasks at 37°C in a 5% CO_2_ atmosphere.

### 4.3. Dissections, immunocytochemistry and TdT-mediated dUTP nick-End-labelling

The dorsal thorax of 20–24 h-old pupae was dissected following the protocol described by Jauffred & Bellaiche [75]. The tissue was fixed in 4% formaldehyde for 20 min, blocked and permeabilized with PBS containing 0.2% BSA, 5% NGS, 0.1% Triton X-100 followed by primary antibody incubation overnight at 4°C and 1 hour secondary antibody incubation at room temperature. Additionally, a DAPI staining was performed together with the secondary antibody incubation. GeneTex FluoroGel mounting media (GeneTex) was used for mounting. Prior to antibody incubations, MCF7 cells were fixed with 3% PFA for 30 min at RT, permeabilized in 0.2% PBS-Triton-X 10 mM Glycine and blocked in 3% BSA.

The following primary antibodies were used to label cell–cell junction proteins: mouse anti E cadherin [1 : 100, abcam (ab1416)]; rat anti-E-Cad [1 : 100, DSHB (DCAD2)], Mouse anti-Armadillo (1 : 100, DSHB), rat anti-alpha-Catenin [1 : 100, DSHB (DCAD1)], Mouse anti-Fasciclin III (1 : 400, DSHB), mouse anti-Coracle [1 : 100, DSHB (C615.16)], mouse anti-DiscsLarge [1 : 100, DSHB (4F3)]; polarity proteins: rat anti-Crumbs (1 : 1000, gift from E. Knust) rabbit anti-Baz (1 : 2,000, gift from A. Wodarz), Rabbit anti-PKC zeta (1 : 50, Santa Cruz); apoptotic cells: rabbit anti-cleaved *Drosophila* Dcp-1 (Asp216, Cell Signalling Technology). Secondary antibodies from Molecular Probes were Alexa Fluor 488, 546 and 633.

Additionally, the ApopTag Red In Situ Apoptosis Detection Kit (S7165, Merck Millipore) was also used to label apoptotic cells following Wells and Johnston protocol [76]. Tissues were incubated as follows: in a pre-cooled ethanol/PBS (2 : 1) solution, 5 min, −20°C; 10 mM Sodium Citrate pH 6.0, 30 min, 70°C; equilibration buffer, 10 min, RT; TdT enzyme (diluted 30% v/v in reaction buffer), 1 h, 37°C; Stop/Wash solution, 10 min, RT; anti-dig rhodamine (20% antibody, 50% PBSt, 25% blocking solution provided with the kit, 5% NGS), 30 min, RT; DAPI (3 mM), 3 min, RT.

Images from fixed samples were acquired on a Zeiss LSM880 inverted confocal microscope using a ×40/1.30 NA oil Ph3 M27 objective, 0.5 µm z-sectioning for polarity and junctional proteins and 1 µm z-sectioning for samples stained for apoptosis.

### 4.4. Live imaging

Using double-sided sticky tape animals with the desired genotype were attached to a custom-made slide which has coverslip bridges on the far edges. A window was then cut in the pupal case and a coverslip with a drop of immersion oil was placed on top of the bridges, touching the notum.

The following inverted confocal microscopes and lenses were used for live imaging: Leica SP2 equipped with a ×40/1.25 NA oil lens; Zeiss LSM880C, ×40/1.30 NA oil Ph3 M27 lens; Zeiss LSM5 Exciter AxioObserver, Plan-Apochromat ×40/1.30 NA oil lens. Samples were excited with a 458 nm laser, and signal was collected at 500–600 nm. Z-series were acquired with a 0.35 µm pixel size using 1 µm z-sectioning. For time-lapses z-series were acquired every 3 min for 2 h.

### 4.5. Transfection

Transfection media containing DharmaFect1 (Dharmacon) transfection reagent and 25 nM ON-TARGET plus SMARTpool siRNA against ING1 (Dharmacon) was added on 50–60% confluent MCF7 cells and 60–80% confluent MDA-MB-231 or U97 cells. ON-TARGETplus Non-targeting siRNA (Dharmacon) was used as a negative control. Cells were then incubated at 37°C with 5% CO2 for 48 h.

### 4.6. Migration and invasion assay

MDA-MB-231, U87 or MCF7 cells were plated in 5% BSA or 1% FBS DMEM or RPMI medium, respectively, in hanging inserts with 8 µm pore (Millipore). Appropriate culture media supplemented with 10% FBS was used underneath the inserts. For invasion experiments, the inserts were covered *a priori* with extracellular matrix (Millipore or Corning). Migrating/ invading cells were detached from the bottom of the inserts using accumax cell detachment solution (Millipore) and stained for DNA content using CyQuant GR dye (Thermo Fisher Scientific). The number of invading cells is direct proportional to the fluorescence intensity as CyQuant dye labels the DNA. The fluorescence intensity was measured using 480/520 nm filters and optimal gain on a BMG fluorescent plate reader.

### 4.7. RNA extraction

Total cellular RNA was extracted using TRIzol (Thermo Fisher Scientific) following the manufacturer's instructions.

### 4.8. cDNA synthesis

cDNA was synthetized using RevertAid First Strand cDNA Synthesis Kit [Thermo Fisher Scientific (k1621)] following the manufacturer's instructions.

### 4.9. Quantitative real-time PCR

Samples were prepared using IQ SYBR Green qPCR Master Mix (BioRad) and non-universal primers (table 1) (Thermofisher Scientific, Sigma-Aldrich or Eurofins) designed using Primer3web online tool. For DNA amplification and fluorescence measurements, an A C1000 thermal cycler CFX96 RT system was used. Relative transcript levels were calculated relative to expression levels of the house-keeping gene GAPDH, using the efficiency calibrated model [77].

**Table 1.** Primer list.

primer name	sequence 5′	sequence 3′
GAPDH human	ATGTTCGTCATGGGTGTGAA	GTCTTCTGGGTGGCAGTGAT
ING1 human	TACTGTCTGTGCAACCAGGT	TTCTCCAGGGCTTTGTCCAT
RHOC human	AGCGGAAGCCCCACCAT	GTCCACCTCAATGTCCGCAA
DIAPH1 human	CGACGGCGGCAAATCTAAGAA	ATTCAGGTTCATATTCCAGCAGCA
ECT2 human	GCAAGAGTGGTTCTGGGGAA	TTGCGATTGCTGTTAGGGGT

### 4.10. Whole-genome gene expression microarray analysis

Whole-genome transcriptome analysis of transfected and un-transfected (both un-treated and treated with non-targeting siRNA) MCF7 cells was conducted at the Nottingham Arabidopsis Stock Centre (NASC). The RNA concentration and quality was assessed using the Agilent 2100 Bioanalyzer (Agilent Technlogies Inc., Palo Alto, CA) and the RNA 600 Nano Kit (Caliper Life Sciences, Mountain View, CA). Samples with a minimum RNA concentration of 100 ng µl^−1^ and RNA Integrity Number (RIN) ≥ 8 were used for gene expression analysis. Single-stranded complimentary DNA was prepared from 200 ng of total RNA as per the GeneChip WT PLUS Reagent Kit (Applied Biosystems and Affymetrix). Total RNA was first converted to cDNA, followed by *in vivo* transcription to make cRNA. Single-stranded cDNA was synthesized, end labelled and hybridized for 16 h at 45°C to Clariom S Assay arrays (Thermo Fisher Scientific).

Gene expression data were analysed using Partek Genomics Suite 6.6 software (Partek Incorporated). The raw CEL files were normalized using the RMA background correction with quantile normalization, log base 2 transformation and mean probe-set summarization with adjustment for GC content. DEGs were identified by a one-way ANOVA. DEGs were considered significant if *p*-value with FDR was ≤ 0.05 and fold change of greater than 1.5 or less than −1.5.

### 4.11. GO term enrichment

DEG following ING1 KD were analysed for biological process and pathway enrichment, using Database for Annotation, Visualization and Integrated Discovery (DAVID) Bioinformatics Resources 6.8 [78]. Functional annotation was performed with EASE score 0.1 and a minimum number of three genes for the corresponding term used as thresholds. Bonferroni correction was applied.

### 4.12. Interaction map

The interaction network was generated by integrating publicly available data from BioGRID using the GeneMANIA plug-in for Cytoscape software [36,79]. GO molecular function was used as weighting for the ties among nodes; 10 max resultant attributes and 20 max resultant genes were used. The resulting network reflects genetic and physical interactions between these genes.

### 4.13. Calculations and statistical analysis

Microsoft Excel and Prism (GraphPad) software were used to perform calculations, generate graphs and calculate statistical significance with Student's *t*-test, Mann–Whitney U test, Kruskall–Wallis test and Pearson Correlation test where *p* > 0.05 was considered not significant, *p < 0.05, **p < 0.01, ***p < 0.001, ^#^*p* < 0.0001 and *r_s_ε*[±0.7, ±1.0] was considered to suggest a strong, *r_s_ε*[±0.5, ±0.7] a moderate, *r_s_ε*[±0.3, ±0.5] a weak and *r_s_ε*[0, ±0.3] a negligible correlation.

For image analysis, standard processing options from Fiji (ImageJ) software were used [80,81]).

## Data accessibility

The accession number for the microarray data reported in this paper is GEO: GSE125438.

The data are provided in the electronic supplementary material [82].
